# Combined mineral-supplemented diet and exercise increases bone mass and strength after eight weeks and maintains increases after eight weeks detraining in adult mice

**DOI:** 10.1371/journal.pone.0204470

**Published:** 2018-09-21

**Authors:** Michael A. Friedman, Robert P. Szczepankiewicz, David H. Kohn

**Affiliations:** 1 Department of Biomedical Engineering, The University of Michigan, Ann Arbor, MI, United States of America; 2 Department of Biologic and Materials Sciences, The University of Michigan, Ann Arbor, MI, United States of America; University of Notre Dame, UNITED STATES

## Abstract

Exercise has long-lasting benefits to bone mass and structural strength even after cessation. Combining exercise with a calcium- and phosphorus-supplemented diet increases cortical bone mineral content (BMC), area, and yield force more than exercise alone in adult mice. These increases could also be maintained after stopping exercise if the modified diet is maintained. It was hypothesized that combining exercise with a mineral-supplemented diet would lead to greater cortical BMC, area, and yield force immediately after a lengthy exercise program and after an equally long period of non-exercise (detraining) in adult mice. Male, 16-week old C57Bl/6 mice were assigned to 9 weight-matched groups–a baseline group, exercise and non-exercise groups fed a control or mineral-supplemented diet for 8 weeks, exercise + detraining and non-exercise groups fed a control or mineral-supplemented diet for 16 weeks. Exercise + detraining consisted of 8 weeks of exercise followed by 8 weeks without exercise. The daily exercise program consisted of running on a treadmill at 12 m/min, 30 min/day. After 8 weeks, mice fed the supplemented diet had greater tibial cortical BMC and area, trabecular bone volume/tissue volume (BV/TV), bone mineral density (vBMD), yield force, and ultimate force than mice fed the control diet. Exercise increased cortical BMC and area only when coupled with the supplemented diet. After 16 weeks, both exercised and non-exercised mice fed the supplemented diet maintained greater tibial cortical BMC and area, trabecular BV/TV, vBMD, yield force, and ultimate force than mice fed the control diet. Combining exercise with a mineral-supplemented diet leads to greater bone mass and structural strength than exercise alone. These benefits remain after an equally long period of detraining. Long-term use of dietary mineral supplements may help increase and maintain bone mass with aging in adult mice.

## Introduction

Weight-bearing exercise offers many benefits to bone health that may help reduce fracture risk. Exercise increases bone mass, structural-level (whole bone) strength, and tissue quality, making bone better able to resist fracture [1]. There is also evidence that exercise has long-term benefits to bone health, even after cessation. Increases in cortical bone mineral content, cross-sectional area, and structural-level strength can remain after months or even years of inactivity following exercise [2–8]. Since bone mass continually declines with age after young adulthood [9–11], it may be beneficial to maximize accumulation of bone mass earlier in life to maintain a higher level of bone mass in old age. Increasing bone mass during older ages may be less likely to occur since bone becomes less responsive to exercise [12,13] and weight-bearing exercise becomes more difficult to perform.

Combining exercise with a calcium- and phosphorus-supplemented diet can be one strategy to maximize bone mass early in adulthood [14]. In young adult mice, combining exercise with a mineral-supplemented diet increases cortical tissue mineral content, area, yield force, and ultimate force more than exercise alone [15]. These differences in bone mass and function appear after 3 weeks of exercise and are maintained or increased when exercise is extended to a duration of 8 weeks. If these increases in bone mass and structural-level strength can also be maintained after stopping exercise, then combining exercise with a mineral-supplemented diet may be a better method of accumulating bone mass earlier in life than just exercise on a standard diet. Thus, it was hypothesized that increases in cortical BMC, area, and structural-level strength after 8 weeks of a combined supplemented diet and exercise program in young adult mice would remain after 8 weeks of detraining. Additionally, it was hypothesized that the combined supplemented diet and exercise treatments would give greater long-term benefits to bone mass and structural-level strength than exercise or diet alone.

## Methods

### Animals and treatments

All animal protocols were approved by the University of Michigan University Committee on Use and Care of Animals. One hundred seventy-six male C57BL/6 mice, 30.2 ± 1.2 g (mean ± SD) body weight, were purchased from Charles River Laboratories (Wilmington, MA) at 14 weeks of age and placed in single housing with enrichment to prevent fighting. The mice were started on the control diet and were given 2 weeks to acclimate. Starting on experiment day 1, at 16 weeks of age, mice were randomly assigned to one of 5 weight-matched groups–a baseline group of 16 mice sacrificed on day 1 (B), a non-exercise group of 40 mice fed the control diet (C), a non-exercise group of 40 mice fed the supplemented diet (D), an exercise group of 40 mice fed the control diet (CE), and an exercise group of 40 mice fed the supplemented diet (DE). After 8 weeks, each experimental group was divided into 2 weight-matched groups of equal number of mice, n = 20 per group. One group was sacrificed immediately (age 24 weeks), and the other group remained part of the experiment for an additional 8 weeks. Mice that continued in the experiment for weeks 9–16 remained on the same diets as during weeks 1–8, but discontinued exercise. The diets remained the same because it would be difficult to differentiate effects of detraining from effects of switching diets. At the end of week 16 (age 32 weeks), all remaining mice were sacrificed. Left tibiae were harvested immediately after sacrifice for analysis. In total, 9 groups were used: baseline (B), no exercise and control diet for 8 weeks (C8), no exercise and supplemented diet for 8 weeks (D8), exercise and control diet for 8 weeks (CE8), exercise and supplemented diet for 8 weeks (DE8), no exercise and control diet for 16 weeks (C16), no exercise and supplemented diet for 16 weeks (D16), exercise + detraining and control diet for 16 weeks (CE16), and exercise + detraining and supplemented diet for 16 weeks (DE16).

### Diets and exercise program

The diets and exercise program were performed as described previously [15]. The control diet contained 0.5% Ca and 0.5% P, and the supplemented diet contained 5% Ca and 1% P. Ca, P, and Ca:P ratio were all increased to increase serum Ca by increasing intestinal Ca absorption [16–18]. The exercise program consisted of running on a 5° incline treadmill at 12 m/min, 30 min/day for 56 consecutive days.

### Cortical geometry and trabecular architecture measurements

Whole tibiae were embedded in 1% agarose, placed in a 19-mm diameter tube, and scanned using a micro-CT specimen scanner (μCT100, Scanco Medical, Bassersdorf, Switzerland) with a voxel size of 12 μm (70 kVp, 114 μA, 0.5 mm AL filter, and integration time 500 ms). Scans were analyzed with Scanco IPL software. A 180-μm thick transverse section from a standard site located 21.7% of the distance from the tibia-fibula junction to the proximal end of the tibia was chosen for measurement of cortical geometry metrics—BMC, volumetric bone mineral density (vBMD), cross-sectional area, and moment of inertia about the anterior-posterior axis. This section is located approximately at the center of the mechanical testing region. Geometry metrics were calculated using a fixed global threshold of 26% (260 on a grayscale of 0–1000) to separate bone from non-bone. Another 180-μm thick transverse section at the fracture site was analyzed for cortical geometry measurements used in calculations of tissue-level mechanical properties (moment of inertia, distance from neutral axis).

Tibial scans were further analyzed for trabecular bone architecture. Proximal tibial metaphyseal sections (480-μm thick) immediately below the growth plate were analyzed in Scanco IPL software using freehand traced volumes of interest. Architecture metrics measured were bone volume (BV), bone volume fraction (BV/TV), volumetric bone mineral density (vBMD), trabecular number (Tb.N), trabecular thickness (Tb.Th), and trabecular separation (Tb.Sp). These metrics were calculated using a fixed global threshold of 18% (180 on a grayscale of 0–1000) to separate bone from non-bone.

### Mechanical testing

After bones were scanned, structural- and tissue-level mechanical properties were measured in all groups as described previously [15]. Structural-level properties (force, deformation, stiffness, work) were measured from a 4-point bending test (3-mm inner and 9-mm outer spans). Tibiae were loaded to failure with the medial side of the mid-diaphysis in tension under displacement control at 0.025 mm/sec with a data sampling rate of 30 Hz. Tissue-level mechanical properties (stress, strain, modulus, toughness) were estimated using beam bending theory with geometric measurements (moment of inertia about anterior-posterior axis, distance from centroid to medial side of the bone) taken from micro-CT data at the fracture site [19].

### Statistical analysis

Cortical geometry measurements, trabecular geometry measurements, and mechanical properties were tested by two-way ANOVA with Tukey’s post-hoc tests to determine if the individual effects of diet or exercise were significant (p < 0.05) and if the combined treatments had a significant interactive effect. Student’s t-tests were used to compare baseline to experimental groups and to compare week 8 to week 16 experimental group values to test effects of detraining and duration of treatments.

## Results

### Mice continuously gained body weight

**Fig 1:**
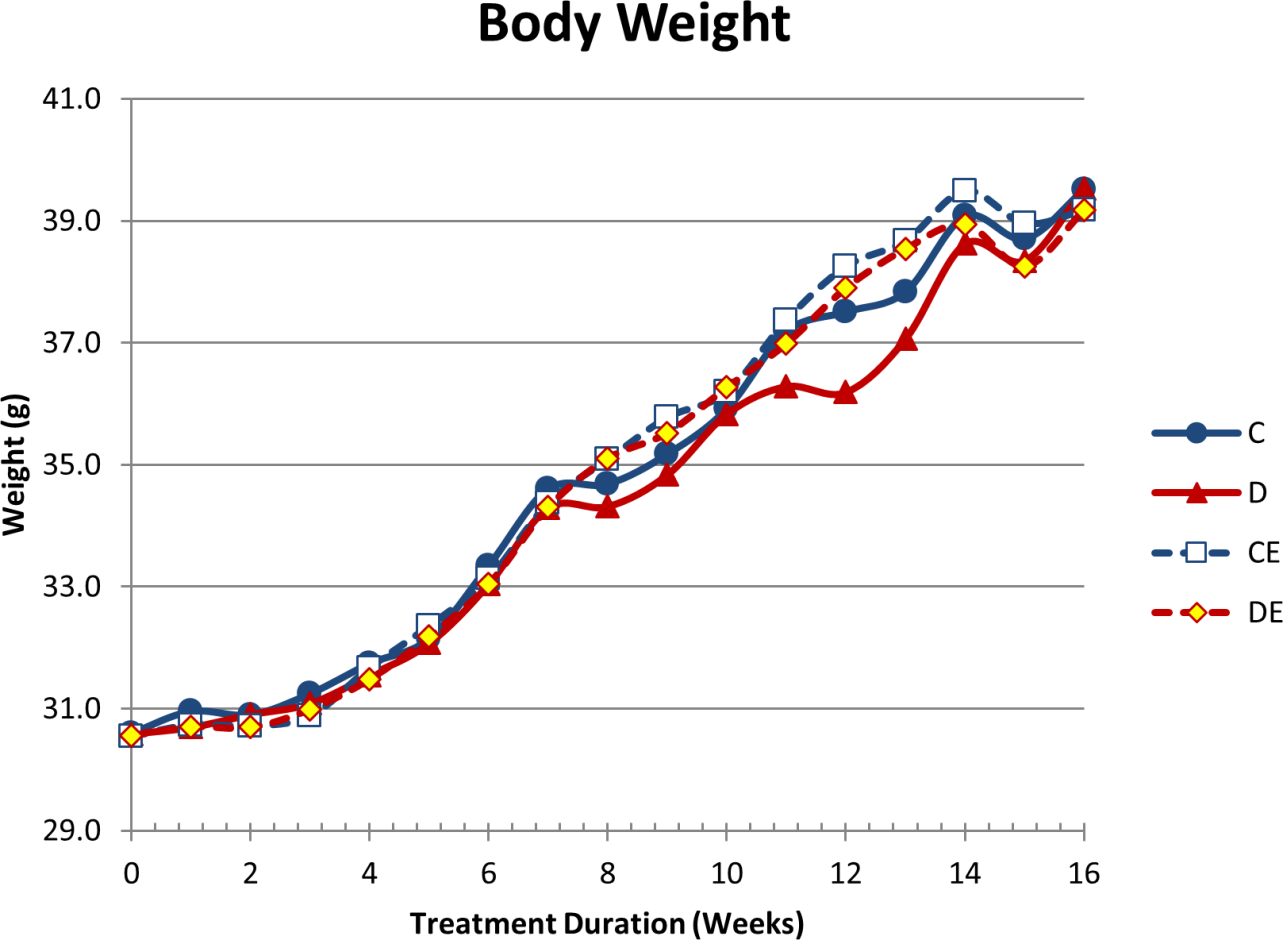
Mouse body weight (mean). All mice started at above average weight for mice of this background strain and gender. Weight increased throughout the study for all groups. There were no significant group differences at any time point. C–non-exercised mice fed the control diet, D–non-exercised mice fed the supplemented diet, CE–exercised mice fed the control diet, DE–exercised mice fed the supplemented diet.

There were no significant differences in mean body weight between any of the groups at any time point (Fig 1). Mice started at an average weight of 30.2 ± 1.4 g (mean ± SD) at 16 weeks of age on day 1. All mice continuously gained weight in the first 14 weeks, ending with an average of 39.4 ± 3.4 g after 16 weeks. The initial and final body weights are above average for male C57BL/6 mice 16 and 32 weeks of age, respectively, and mice had an above average rate of weight gain during this study [20,21].

### Exercise on the supplemented diet increased tibial cortical bone mass after eight weeks

**Fig 2:**
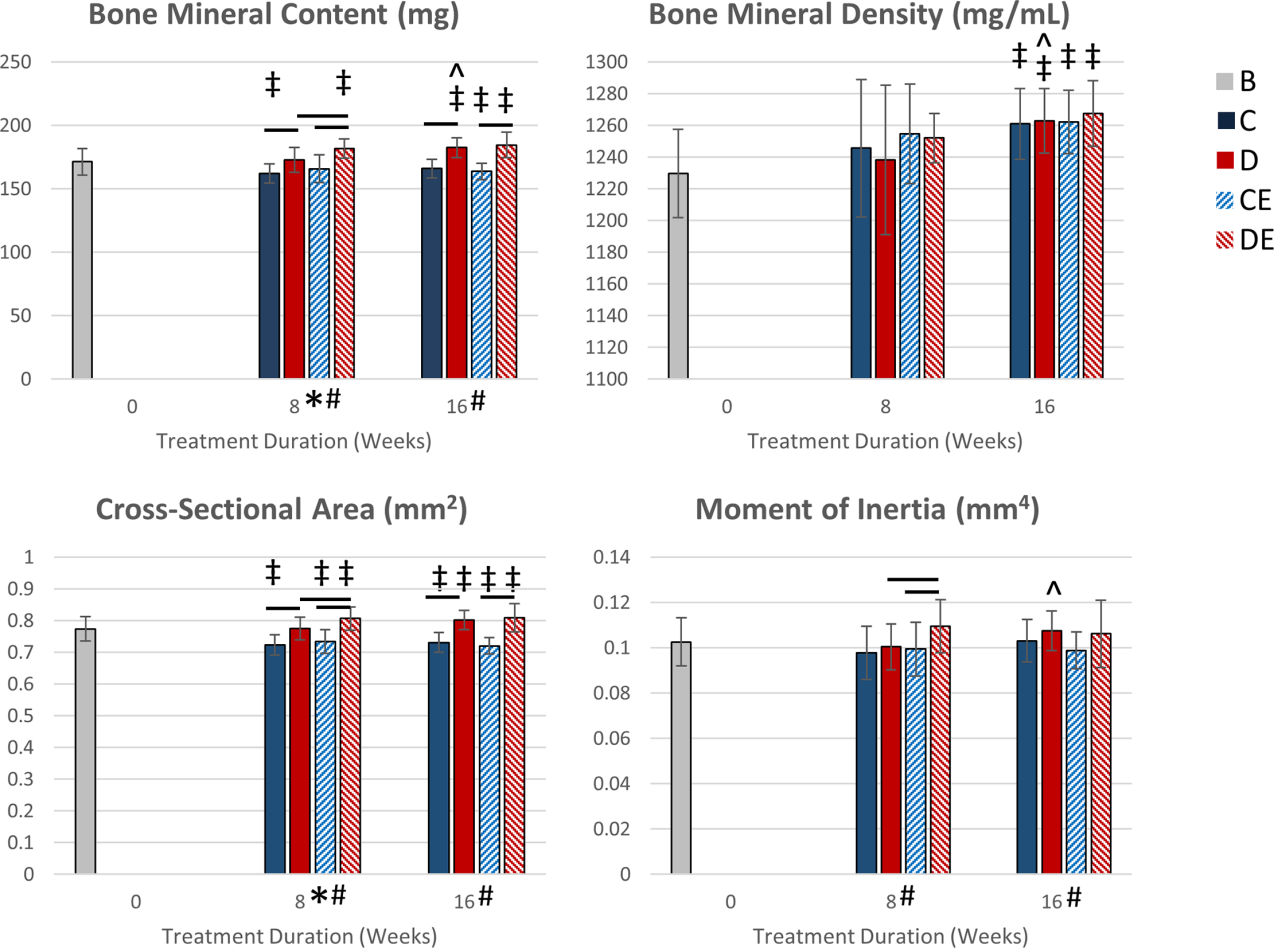
Tibial cortical cross-sectional geometric properties (mean ± SD). After 8 weeks of exercise, DE8 mice had the greatest BMC, area and MoI. After 8 weeks, there was a significant main effect of diet on BMC, area, and moment of inertia, and there was a significant main effect of exercise on BMC and area. After 16 weeks (8 exercise + 8 detraining), there was a significant main effect of diet on BMC, area, and MoI. D8 mice had no change in bone mass from baseline to 8 weeks. D16 mice had greater cortical area and BMC than D8 mice. C8 and C16 mice had lower area than baseline at both time points. *Significant exercise effect (p < 0.05, Two-way ANOVA) #Significant diet effect (p < 0.05, Two-way ANOVA) -Significant group difference (p < 0.05, Tukey’s test) ‡Significantly different from baseline (p < 0.05, t-test) ^Significantly different from after 8 weeks (p < 0.05, t-test) B—baseline mice, C–non-exercised mice fed the control diet, D–non-exercised mice fed the supplemented diet, CE–exercised mice fed the control diet, DE–exercised mice fed the supplemented diet.

Exercise had a significant main effect on cortical BMC and cross-sectional area after 8 weeks (p < 0.05, Two-way ANOVA, Fig 2). Diet had a significant main effect on cortical BMC, area, and moment of inertia about the anterior-posterior axis. In DE8 mice, eight weeks of exercise on the supplemented diet led to significantly greater cortical BMC, area, and moment of inertia compared to CE8 and D8 mice (p < 0.05, Tukey’s tests). BMC and area significantly increased from baseline only in DE8 mice (p < 0.05, t-test). CE8 mice had significantly decreased cortical area from baseline. C8 mice had significantly decreased cortical BMC and area from baseline. The supplemented diet prevented these decreases in D8 mice. vBMD significantly increased from baseline in both CE8 and DE8 mice.

### Exercise on the supplemented diet increased tibial trabecular bone volume after eight weeks

**Fig 3:**
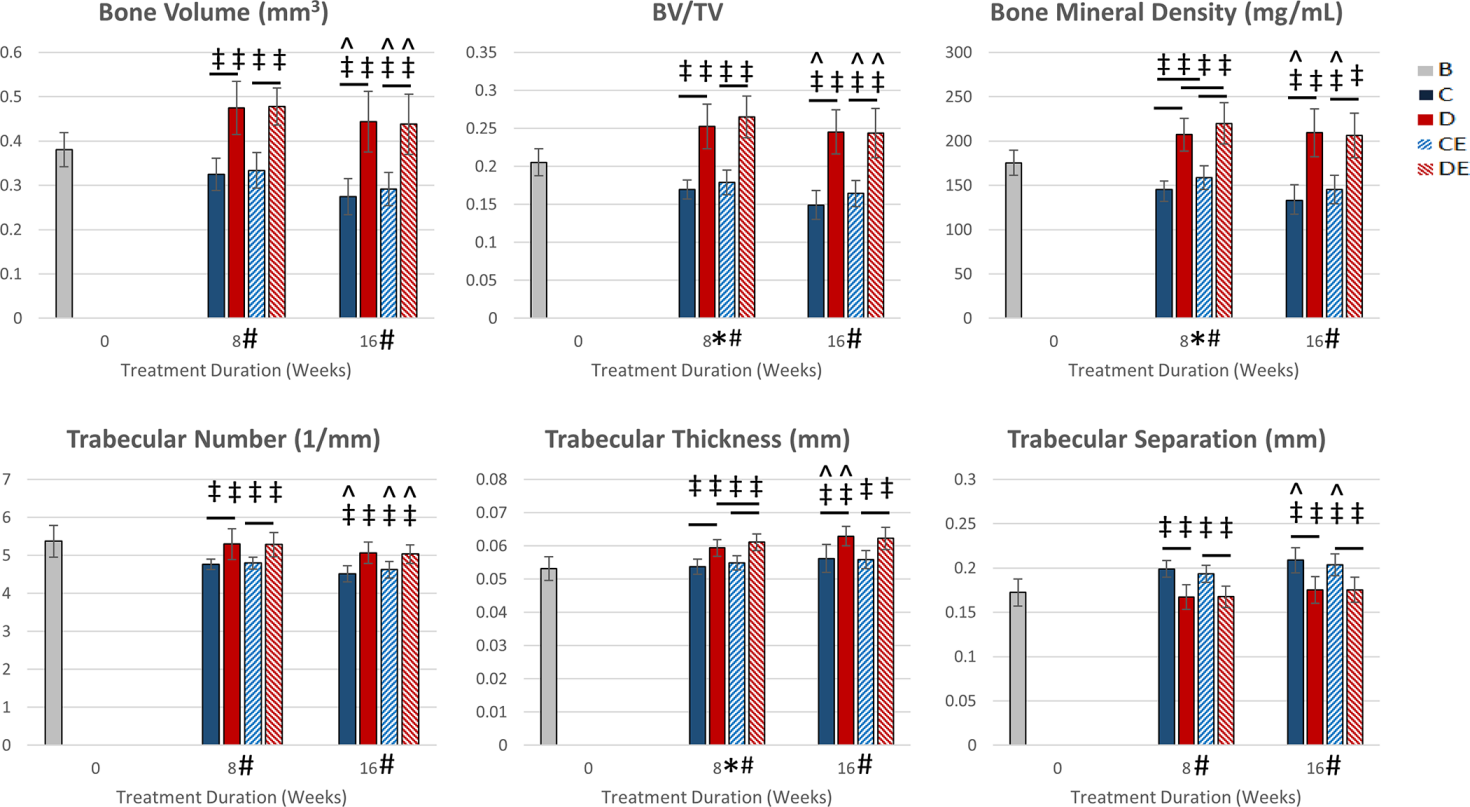
Proximal tibial trabecular architecture measures (mean ± SD). After 8 weeks, there was a significant main effect of exercise on BV/TV, BMD, and Tb. Th, and there was a significant main effect of diet on bone volume, BV/TV, BMD, Tb. N, Tb. Th, and Tb. Sp. In C8 and CE8 mice, the control diet decreased most measurements of trabecular architecture from baseline. Conversely, in D8 and DE8 mice, the supplemented diet prevented decreases or increased trabecular architecture measurements from baseline. After 16 weeks (8 exercise + 8 detraining), there was a significant main effect of diet on bone volume, BV/TV, BMD, Tb. N, Tb. Th, and Tb. Sp. The D16 mice maintained BV, BV/TV, and Tb.N while all other groups had lower values than after 8 weeks. *Significant exercise effect (p < 0.05, Two-way ANOVA) #Significant diet effect (p < 0.05, Two-way ANOVA) -Significant group difference (p < 0.05, Tukey’s test) ‡Significantly different from baseline (p < 0.05, t-test) ^Significantly different from after 8 weeks (p < 0.05, t-test) B—baseline mice, C–non-exercised mice fed the control diet, D–non-exercised mice fed the supplemented diet, CE–exercised mice fed the control diet, DE–exercised mice fed the supplemented diet.

Exercise had a significant main effect on trabecular BV/TV, vBMD, and Tb.Th after 8 weeks (p < 0.05, Two-way ANOVA, Fig 3). Diet had a significant main effect on trabecular BV, BV/TV, vBMD, Tb.N, Tb.Th, and Tb.Sp. DE8 mice had significantly greater BV, BV/TV, vBMD, Tb.N, Tb.Th and significantly less Tb.Sp than CE8mice (p < 0.05, Tukey’s tests). D8 and DE8 mice had significantly greater BV, BV/TV, vBMD, and Tb.Th than baseline mice (p < 0.05, t-test). The control diet had the opposite effect, as C8 and CE8 mice had significantly lower BV, BV/TV, vBMD, Tb.N and significantly greater Tb.Sp than baseline. CE8 and DE8 mice had significantly greater vBMD than C8 and D8 mice, respectively.

### Exercise on the supplemented diet increased tibial structural-level strength after eight weeks

**Fig 4:**
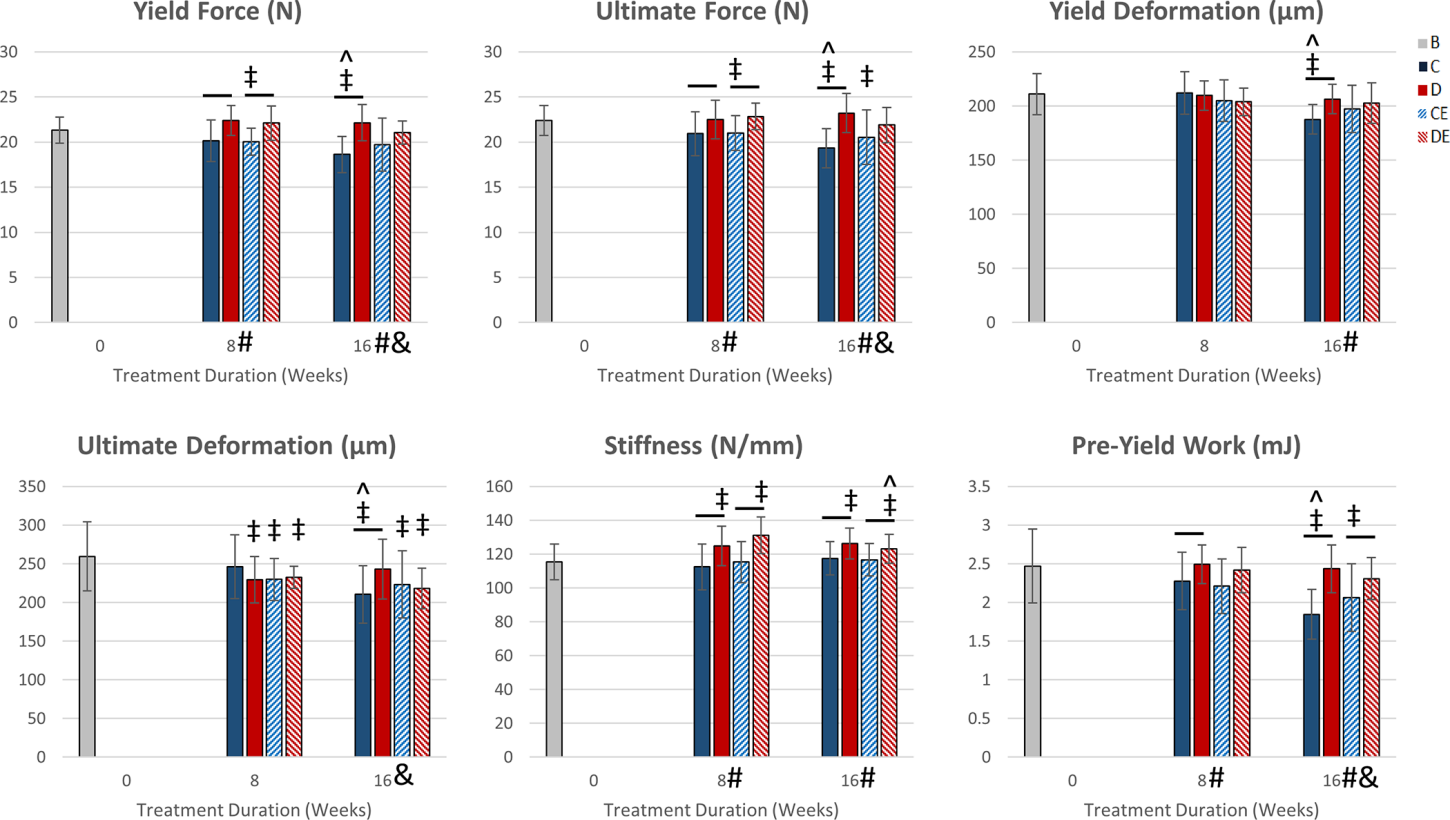
Structural-level tibial mechanical properties (mean ± SD). After 8 weeks, there was a significant main effect of diet on yield force, ultimate force, stiffness, and pre-yield work. D8 and DE8 mice had significantly greater tibial yield force, ultimate force, and stiffness than C8 and CE8 mice, respectively. After 16 weeks (8 exercise + 8 detraining), there was a significant diet and exercise interaction on yield force, ultimate force, ultimate deformation, and pre-yield work. C16 mice had decreased structural-level strength from 8 to 16 weeks. Exercise and the supplemented diet prevented these decreases in the other groups. *Significant exercise effect (p < 0.05, Two-way ANOVA) #Significant diet effect (p < 0.05, Two-way ANOVA) &Significant diet and exercise interaction (p < 0.05, Two-way ANOVA) -Significant group difference (p < 0.05, Tukey’s test) ‡Significantly different from baseline (p < 0.05, t-test) ^Significantly different from after 8 weeks (p < 0.05, t-test) B—baseline mice, C–non-exercised mice fed the control diet, D–non-exercised mice fed the supplemented diet, CE–exercised mice fed the control diet, DE–exercised mice fed the supplemented diet.

Exercise had no significant main effects on structural-level mechanical properties after 8 weeks (p < 0.05, Two-way ANOVA, Fig 4). Diet had a significant main effect on yield force, ultimate force, stiffness, and pre-yield work. DE8 mice had significantly greater tibial yield force, ultimate force, and stiffness than CE8 mice (p < 0.05, Tukey’s tests). D8 mice had significantly greater tibial yield force, ultimate force, stiffness, and pre-yield work than C8 mice. Both D8 and DE8 mice had significantly greater tibial stiffness and significantly lower ultimate deformation than baseline mice (p < 0.05, t-test). CE8 mice also had significantly lower tibial yield force, ultimate force, and ultimate deformation than baseline mice.

### Diet and exercise and did not affect tibial tissue-level mechanical properties after eight weeks

**Fig 5:**
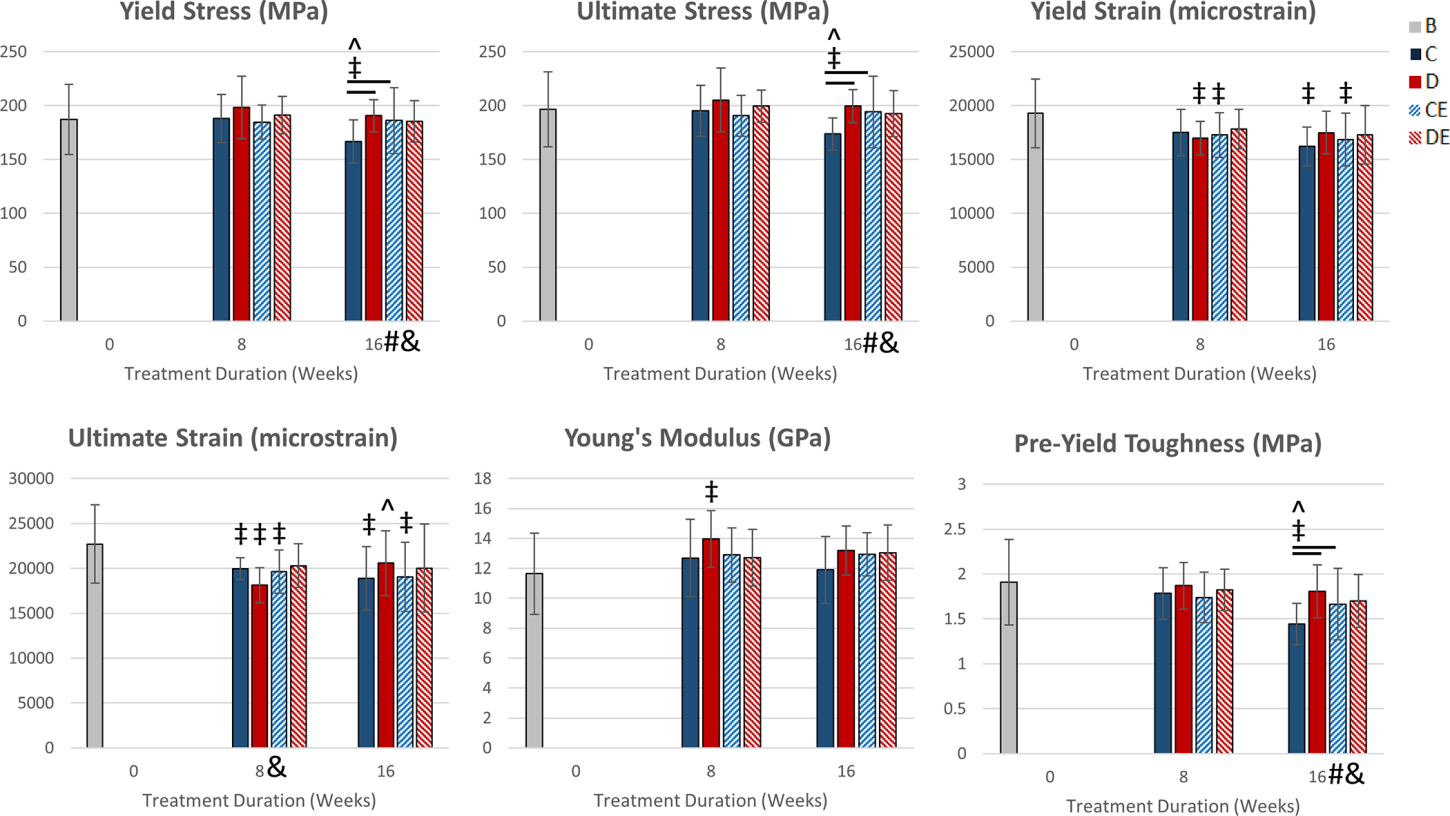
Tissue-level tibial mechanical properties (mean ± SD). After 8 weeks, there was a significant diet and exercise interaction on ultimate strain. After 16 weeks (8 exercise + 8 detraining), there was a significant diet and exercise interaction on yield stress, ultimate stress, and pre-yield toughness. There was decreased yield stress, ultimate stress, and pre-yield toughness in the C16 group, compared to the C8 group. Both exercise and the supplemented diet prevented this decrease in strength. *Significant exercise effect (p < 0.05, Two-way ANOVA) #Significant diet effect (p < 0.05, Two-way ANOVA) &Significant diet and exercise interaction (p < 0.05, Two-way ANOVA) -Significant group difference (p < 0.05, Tukey’s test) ‡Significantly different from baseline (p < 0.05, t-test) ^Significantly different from after 8 weeks (p < 0.05, t-test) B—baseline mice, C–non-exercised mice fed the control diet, D–non-exercised mice fed the supplemented diet, CE–exercised mice fed the control diet, DE–exercised mice fed the supplemented diet.

There were no significant main effects of diet or exercise on any tissue-level mechanical property, but there was a significant diet and exercise interaction on ultimate strain after 8 weeks (p < 0.05, Two-way ANOVA, Fig 5). There were also no significant group differences for any tissue-level mechanical property measured after 8 weeks. CE8 mice had significantly lower yield strain and ultimate strain than baseline mice (p < 0.05, t-test). D8 mice had significantly greater Young’s modulus and significantly lower yield strain and ultimate strain than baseline mice. C8 mice had significantly lower ultimate strain than baseline mice. C8 mice had significantly lower ultimate strain than baseline mice.

### Eight weeks of detraining maintained tibial cortical geometry in exercised mice while the supplemented diet increased tibial cortical bone mass in non-exercised mice

After 8 weeks of detraining (defined here and in Figures as 16 weeks for brevity), exercise had no significant main effects (p < 0.05, Two-way ANOVA, Fig 2). Diet had significant main effects on cortical BMC, area, and moment of inertia. D16 mice on had significantly greater BMC, vBMD, area, and moment of inertia than D8 mice (p < 0.05, t-test). vBMD was significantly greater than baseline in all groups after 16 weeks (p < 0.05, t-test), and there were no significant differences in vBMD between the 16-week groups. There were no significant decreases in cortical bone cross-sectional geometry measurements from 8 to 16 weeks.

### Effects of diet on tibial trabecular architecture were maintained after detraining

Exercise had no significant main effects on trabecular architecture after detraining (p < 0.05, Two-way ANOVA, Fig 3). Diet had significant main effects on BV, BV/TV, vBMD, Tb.N, Tb.Th, and Tb.Sp after detraining. After detraining, DE16 mice had significantly greater BV, BV/TV, vBMD, Tb.Th and significantly lower Tb.Sp than CE16 mice (p < 0.05, Tukey’s tests). Both D16 and DE16 mice had significantly greater BV, BV/TV, vBMD, and Tb.Th than baseline mice (p < 0.05, t-tests). The control diet had the opposite effect after 16 weeks as both C16 and CE16 mice had significantly lower BV, BV/TV, vBMD, and Tb.N and significantly greater Tb.Sp than baseline mice. Tb.Th was significantly increased from baseline for all groups and greater than it was after 8 weeks for the C16 and D16 mice. C16 and CE16 mice had significantly greater Tb.Sp than C8 and CE8 mice, respectively. For C16, CE16, and DE16 mice, BV, BV/TV, and Tb.N were significantly lower after 16 weeks than after 8 weeks (p < 0.05, t-tests). After 16 weeks, vBMD in C16 mice was significantly lower than vBMD in C8 mice.

### Diet and exercise had a significant interactive effect on tibial structural-level strength after 8 weeks of detraining

Diet and exercise had a significant interaction on yield force, ultimate force, ultimate deformation, and pre-yield work after detraining (p < 0.05, Two-way ANOVA, Fig 4). Exercise had no significant main effects on tibial structural strength measurements after detraining. DE16 mice had significantly greater stiffness and pre-yield work than CE16 mice after 8 weeks of exercise plus 8 weeks detraining (p < 0.05, Tukey’s tests). D16 mice had significantly greater yield force, ultimate force, yield deformation, ultimate deformation, stiffness, and pre-yield work than C16 mice. C16 mice had significantly lower yield force, ultimate force, yield deformation, ultimate deformation, and pre-yield work than C8 mice (p < 0.05, t-test). CE16 mice did not show this decline from CE8 mice even though exercise was discontinued after 8 weeks.

### Diet and exercise had a significant interactive effect on tibial tissue-level strength after 8 weeks of detraining

Diet and exercise had a significant interaction on yield stress, ultimate stress, and pre-yield toughness after detraining (p < 0.05, Two-way ANOVA, Fig 5). Exercise had no significant main effects on tissue strength measurements after detraining. CE16 mice and D16 mice had significantly greater yield stress, ultimate stress, and pre-yield toughness than C16 mice (p < 0.05, Tukey’s tests). For C16 mice, these tissue-level mechanical properties were all significantly lower than in C8 mice. CE16 mice did not show this decline from CE8 mice even though exercise was discontinued after 8 weeks.

## Discussion

Long-term consumption of the mineral-supplemented diet increased and maintained bone mass and bone strength in DE16 mice, even after detraining. Eight weeks of exercise in conjunction with a mineral-supplemented diet led to significantly greater tibial cortical BMC, area, and moment of inertia in DE8 mice compared to D8 and CE8 mice (Fig 2). These cross-sectional properties significantly increased from 8 to 16 weeks in D16 mice such that there were no differences in cortical bone measurements between D16 and DE16 mice. Thus, the data suggest combining exercise with the supplemented diet leads to peak cortical bone mass at a faster rate than with the supplemented diet without exercise. Exercise had little effect on cortical bone mass in mice fed the control diet, as there were no significant differences in cross-sectional properties between exercised and non-exercised mice on the control diet at either 8 or 16 weeks.

As with cortical bone, diet was more impactful on trabecular architecture than exercise (Fig 3). The cortical bone data suggests both exercised and non-exercised mice on the supplemented diet achieved the same peak bone mass but at different rates, and a similar trend may occur with trabecular bone volume, but at a different time. Since trabecular bone has more rapid turnover, this may have led to the supplemented diet groups reaching peak BV, BV/TV, vBMD, and Tb.N by 8 weeks.

As was done for our previous study [15], dietary Ca was increased to provide similar benefits as when it was used in ovariectomized mice [22]. Fonseca and Ward used a supplemented diet with 12.5X Ca of the control diet. We observed no negative health effects from using the supplemented diet with 10X Ca of the control diet here and for 8 weeks in our previous study. There are drastic differences between human and mouse Ca requirements and consumption. Differences in dietary requirements and compositions make it difficult to directly translate Ca requirements from mouse diets to human diets. Studies evaluating increases in dietary mineral in rodents have largely only increased Ca beyond the required amount [23]. There is evidence that also increasing dietary phosphorus [16] and Ca:P ratio [17] could be beneficial to bone mass and strength. In our studies, there appears to have been some added benefits to bone mass and strength from increasing both Ca and P that was not seen in other studies that only increased Ca.

Diet was more impactful than exercise as diet had more significant main effects and greater magnitudes of effects. This is different than previous work showing exercise to be more beneficial [24]. Welch *et al*. examined normal Ca intake (0.5%) vs insufficient intake (0.2%). We examined the effects of supplementing Ca beyond what is normally required in adult mice. Exercise is expected to be most beneficial during skeletal development, leading to increased bone mass, strength, and tissue quality. In adults, exercise is not expected to increase bone mass, but it is still beneficial for bone strength and tissue quality.

After detraining, differences in cortical and trabecular bone geometry between the two exercised groups remained (Figs 2–3). This was likely due to maintaining the mice on the mineral-supplemented diet, as differences in bone geometry between the non-exercised groups also remained after 16 weeks on the diets. The mineral-supplemented diet played a stronger role than exercise and detraining in determining cortical and trabecular bone mass. For mice on the supplemented diet, during detraining cortical BMC and area was maintained in exercised mice while non-exercised mice increased BMC and area to the levels of the exercised mice.

The supplemented diet increased structural-level strength as there were significant main effects of diet on yield force, ultimate force, stiffness, and pre-yield work after 8 weeks (Fig 4). These increases are to be expected since the supplemented diet also significantly increased cortical bone area and BMC (Fig 2). For DE8 mice, 8 weeks of exercise did not increase structural strength beyond what was achieved in D8 mice, despite significantly increasing cortical BMC and area. It may be possible that the greater cortical bone mass in the DE8 mice consists of tissue having a composition that does not increase structural-level strength.

After detraining, the magnitude of the differences in structural-level mechanical properties between the CE and DE mice decreased such that there was no longer a significant difference in yield force and ultimate force (Fig 4). It is possible that exercise-induced adaptation of the tibia was still occurring after exercise stopped, preventing a loss of strength in CE16 mice. Bone can still adapt to exercise after loading stops, leading to improvements not noticed if mice are sacrificed immediately at end of the exercise program [25,26]. For CE8 mice, there were no significant differences in tissue-level mechanical properties after 8 weeks, compared to C8 mice. CE16 mice exhibited significantly greater yield stress, ultimate stress, and pre-yield toughness than C16 mice after 8 weeks detraining (Fig 5). Although exercise did not prevent a decline in structural-level strength from baseline to 8 weeks for CE8 mice (Fig 4), exercise did prevent a decrease in structural-level and tissue-level strength after 8 weeks of detraining for CE16 mice. This occurred even though the CE16 mice had the same activity level as the C16 mice during that time. Exercise increases cortical bone tissue quality without increasing bone mass, and that may be what caused bones from CE16 mice to have greater mechanical strength after 16 weeks without having greater cortical bone mass [27]. For CE16 mice, exercise had a long-lasting effect in preventing loss of bone strength from 8 to 16 weeks.

Exercise did not increase cortical bone mass after 8 weeks as it did in our prior experiments using this model [15]. Lack of exercise effects may have been due to the higher mouse body weights initially and throughout the study. Greater body weight may have caused greater loading on bones during daily cage activities, making the exercise loads less conducive to adaptation since the bones may have already been subjected to loads high enough for peak osteogenic effects. The high body weight of the mice may also play a role in preventing exercise from affecting trabecular bone. Also, the timing of when the mice were sacrificed may be more in line with when cortical bone effects are significant but not trabecular bone effects. Unlike our prior experiments [15], in this study, the mice were singly housed. Singly-housed mice have lower cortical BMC, which can result in decreased structural-level strength [28]. Single housing also could have been a factor in the lack of exercise effects on cortical bone.

C8 and CE8 mice had signs of age-related bone loss as cortical area (Fig 2), BV, BV/TV, vBMD, and Tb.N (Fig 3) were significantly lower than baseline after 8 weeks. Whereas cortical bone properties plateaued at 8 weeks, trabecular bone properties declined between 8 and 16 weeks. These changes in bone geometry are consistent with age-related changes shown in adult male C57Bl/6 mice after 3–5 months of age [29]. Age-related loss of cortical and trabecular bone mass in mice fed the control diet was prevented or reversed in mice fed the mineral-supplemented diet. Increased bone mass from the supplemented diet came at no expense to structural- or tissue-level strength. Thus, the standard rodent diet may not be ideal for maximizing bone mass in adult male C57Bl/6 mice in studies involving interventions that affect bone health.

Large increases in body weight also may have contributed to decreases in cortical and trabecular bone mass as well as decreases in structural- and tissue-level strength in mice fed the control diet, since obesity can decrease bone mass and strength [30,31]. The diets used may have been a factor in the weight gain. Although the diets were not high in fat (16.3% calories from fat), the high carbohydrate content (65% calories from carbohydrates) may have caused excessive weight gain. Adding Ca supplements to high-fat diets prevents loss of cortical bone mineral content in obese mice [32]. If the diets used in this study did induce obesity, causing negative effects on bone mass and bone strength, the supplemented diet may have helped to prevent or attenuate some of the detrimental effects.

In this study, detraining maintained almost all effects on bone mass and bone strength. To our knowledge, this is the first study to show that long-term consumption of a mineral-supplemented diet leads to increases and maintenance of cortical bone, trabecular bone, structural-level strength, and tissue-level strength in adult mice. These increases occurred without detrimental effects on bone health. The data suggests long-term consumption of a mineral-supplemented diet may be beneficial in preventing loss of structural- and tissue-level strength with age. Exercise was also beneficial as it allowed mice on the supplemented diet to achieve peak cortical bone mass in less time and prevented loss of trabecular bone and loss of bone strength from 8 to 16 weeks in mice on the control diet. Long-term use of dietary mineral supplements may help increase and maintain bone mass with weight gain and/or aging in adult mice.

## Supporting information

S1 Dataset Raw data for body weight, cortical bone, trabecular bone, and mechanical properties. (XLSX)
